# Stigma of People with HIV/AIDS in Sub-Saharan Africa: A Literature Review

**DOI:** 10.1155/2009/145891

**Published:** 2009-08-16

**Authors:** Ngozi C. Mbonu, Bart van den Borne, Nanne K. De Vries

**Affiliations:** Department of Health Promotion, School of Public Health and Primary Care CAPHRI, Faculty of Health, Medicine and Life Sciences, Maastricht University, P.O. Box 616, 6200 MD Maastricht, The Netherlands

## Abstract

The aim of this literature review is to elucidate what is known about HIV/AIDS and stigma in Sub-Saharan Africa. Literature about HIV/AIDS and stigma in Sub-Saharan Africa was systematically searched in Pubmed, Medscape, and Psycinfo up to March 31, 2009. No starting date limit was specified. The material was analyzed using Gilmore and Somerville's (1994) four processes of stigmatizing responses: the definition of the problem HIV/AIDS, identification of people living with HIV/AIDS (PLWHA), linking HIV/AIDS to immorality and other negative characteristics, and finally behavioural consequences of stigma (distancing, isolation, discrimination in care). It was found that the cultural construction of HIV/AIDS, based on beliefs about contamination, sexuality, and religion, plays a crucial role and contributes to the strength of distancing reactions and discrimination in society. Stigma prevents the delivery of effective social and medical care (including taking antiretroviral therapy) and also enhances the number of HIV infections. More qualitative studies on HIV/AIDS stigma including stigma in health care institutions in Sub-Saharan Africa are recommended.

## 1. Introduction

Although the current data show that the global HIV/AIDS epidemic is stabilizing, statistics still report an unacceptably high level of infection and progress is uneven in many countries [1]. In 2007, approximately 33 million people worldwide were infected with the human immunodeficiency virus (HIV) [1]. Sub-Saharan Africa remains the most affected region in the world and it is home to almost 67% of all people living with HIV (an estimated 22.5 million [1]). In 2007, an estimated 1.7 million adults and children in this region became newly infected, while 1.6 million died of acquired immune deficiency syndrome (AIDS). 

People living with HIV/AIDS (PLWHA) face not only medical problems but also social problems associated with the disease. One of the barriers to reaching those who are at risk or infected with HIV/AIDS is stigma [2]. Stigma enhances secrecy and denial, which are also catalysts for HIV transmission [3]. Although the reaction to PLWHA varies, with some PLWHA receiving support which positively affects them, HIV/AIDS stigma negatively affects seeking HIV testing, seeking care after diagnosis, quality of care given to HIV patients, and finally the negative perception and treatment of PLWHA by their communities and families, including partners [4, 5]. It isolates people from the community and affects the overall quality of life of HIV patients [2, 3, 6, 7]. 

Currently, there is an increasing number of research on HIV-related stigma in Sub-Saharan Africa. It is being increasingly acknowledged, however, that effective treatment and care strategies require an understanding of the cultural context [6, 8] in which stigma exists. The aim of this literature review is to elucidate what is known about HIV/AIDS stigma in Sub-Saharan Africa, the origins and contents of stigma, contributing factors and the gender dimension of stigma.

## 2. Methodology

The analyses in this review paper were based on Gilmore and Somerville's [9] classification of stigmatization in sexually-transmitted diseases, which for this paper was applied to the various factors that affect stigmatization of PLWHA. 

## 3. Materials

Literature about HIV/AIDS stigma in Sub-Saharan Africa was systematically searched in Pubmed, Medscape, and Psycinfo up to March 31, 2009. No starting date was specified. 

A first search in Medline, PsycInfo and Pubmed with “HIV/AIDS”, “stigma” and “Africa” as key words gave 292 abstracts, of which 91 abstracts came from Medline, 57 from PsycInfo, and 144 from Pubmed; another search with “HIV/AIDS”, “discrimination” and “Africa” gave an additional 192 abstracts (Medline 73, PsycInfo 15, and Pubmed 104). A total of 484 abstracts were examined. From this list, papers relevant to the aim of this review were selected on the basis of their abstracts; uncertainties were reconciled through discussion with all the authors of this paper. The completeness of the search was checked by means of the reference lists of reviewed articles. Books or book chapters were included whenever applicable. Exclusion criteria included newspaper articles, campaign posters, articles not in the English language and articles not related to the topic. On the basis of this selection, 64 original articles were critically evaluated.

## 4. Outcome Selection

HIV stigma as a phenomenon was considered to be the major topic of the review and was not limited to any geographical region. Although HIV/AIDS stigma is a general phenomenon which affects PLWHA in all parts of the world, in this paper, we focused on an analysis of the factors contributing to stigma identified from empirical studies in Sub-Saharan Africa, books, theoretical, and review papers. They include cultural constructions, stereotypes and specific beliefs, access to and the role of antiretroviral therapy, religion, and gender.

## 5. Results and Discussion

**Figure 1:**
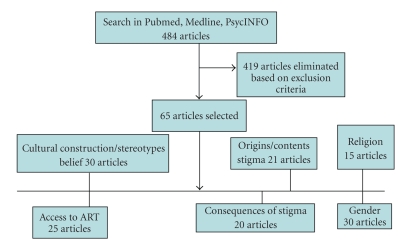


Figure 1 shows the flow chart of the search results. A total of 64 articles were selected. Twenty of these articles were theoretical papers, review papers and articles on stigma not limited to any geographical location while 45 articles were empirical studies from Sub-Saharan Africa. For the analysis of the contributing factors to HIV/AIDS stigma, 45 articles from empirical studies within Sub-Saharan Africa were used. In addition, books, theoretical papers and review papers were also used. Table 1 shows the method, study objective, study population and country of study of these papers; 21 articles addressed origin and contents of stigma, 30 articles addressed cultural constructions of HIV, stereotypes and specific beliefs, 25 articles addressed access to and the role of ART, 16 articles addressed religion, 30 articles addressed gender and 20 articles addressed consequences of stigma. 

## 6. Origin and Content of Stigma

Etymologically, the concept of “stigma” derives from a Greek word referring to a tattoo mark. It generally has two meanings. One derived from Christianity and denotes bodily marks which resemble those of the crucifixion of Jesus Christ—they are attributed to divine favour. The second meaning is secular, namely marks of disgrace, discredit, or infamy [9]. Today, the term “stigma” is applied more to social disgrace than to any bodily signs [10]. Stigma is generally recognized as an “attribute that is deeply discrediting” that reduces the bearer “from a whole and usual person to a tainted, discounted one” [11]. Stigma is also used to set the affected persons or groups apart from the normalized social order (“us” against “them”) and this separation implies devaluation [2, 9, 12, 13]. HIV stigma is shaped not only by individual perceptions and interpretations of microlevel interactions but also by larger social and economic forces [6]. It is a social construct, which has significant impact on the life experiences of individuals both infected and affected by HIV [14]. Stigma includes prejudice and can lead to active discrimination directed toward persons either perceived to be or actually infected with HIV and the social groups and persons with whom they are associated [15]. Since not all stigmatizing attitudes result in overtly discriminatory behaviors, Campbell et al. [6] described discrimination as negative behavior and stigmatization as any negative thoughts, feelings, or actions toward PLWHA irrespective of whether people are discriminated because they know that they are devalued. In other words, discrimination has to be acted out externally while stigmatization can be overt or constitute libel, slander, or defamation of persons who are stigmatized [9]. 

Stigma can be external or internal [3]. External stigma refers to the actual experience of discrimination [57]. Internal stigma (felt or imagined stigma) is the shame associated with HIV/AIDS and PLWHAs' fear of being discriminated against [2, 58]. Internal stigma is a powerful survival mechanism aimed at protecting oneself from external stigma and often results in thoughts or behavior such as the refusal or reluctance to disclose a positive HIV status, denial of HIV/AIDS and unwillingness to accept help [2, 7, 16, 17]. This collective public denial in societies is reflected by avoidance of mentioning any terminal illness including HIV/AIDS, a need to keep hope alive for therapeutic success, stigma attached to HIV/AIDS, and unwillingness to confront matters related to sexuality [17]. Many authors have theorized and produced models of stigmatization, but this paper will apply Gilmore and Somerville's [9] categorization of stigmatization in sexually transmitted diseases. They argued that any stigmatizing response has at least four processes [9].

1. *The problem*. The problem (in the context of this paper, HIV/AIDS) which Goffman [11] describes as the discredited attribute and Link and Phelan [59] describe in the component of conceptualizing stigma as distinguishing and labellizing, has to be such that the response in some way permits the stigmatizer to be spared, saved, or gives power to control the problem.
2. *Identification of the person or group who are targeted for stigmatization*. This means PLWHA must be recognizable and therefore have some identifying characteristics that can be used to recognise them correctly or erroneously, for example, loss of weight, skin rash, and so forth [2, 7, 8, 17–19, 60]. This process was also described by Link and Phelan [59] in the component of conceptualizing stigma as distinguishing and labellizing.
3. *Application of stigma to the target person*. Here specific persons are labelled with stigma. The stigma and the negative characteristics associated with it are perceived as belonging to them, for example, someone who is stigmatized is perceived as immoral [2, 6, 20]. This according to Link and Phelan [59] in the component of conceptualizing stigma relates to negative stereotyping.
4. *The outcome is usually a response to the stigmatized person such that they are distanced, disempowered or controlled by the stigmatizer*. In this process, there is a change in the relationship or interaction between stigmatizer and the stigmatized [6].

In this literature review these four processes of stigmatization will be illustrated in an analysis of the various factors that affect HIV/AIDS stigma. 

## 7. Factors in HIV/AIDS Stigma

Everywhere HIV/AIDS has been accompanied by stigma and discrimination but stigma in Sub-Saharan Africa seems to be particularly common [2, 7, 55]. What happens to one person concerns the whole community [2, 3, 6, 7, 17, 21, 22, 61]. The communal life in itself poses a dilemma because, on the one hand, it can bring about stigmatization when PLWHA are not able to interact owing to fear of being exposed [2, 6, 13, 17, 21, 23, 48, 55], but, on the other hand, communal life also ensures help and care for sick people [2, 6, 7, 21, 22, 24]. It is important to understand how stigma is used by individuals and communities, in cultures where communal life is common, to produce and reproduce inequality [62]. Stigmatization is part of a conservative reassertion of power relations, poverty, or moral authority resting on the ability to control sexuality [6]. Because PLWHA are labelled as the “other” by the community, people try to secure the social structure, safety and solidarity by casting out offenders or reaffirming societal values [9]. PLWHA are assumed not to be able to contribute to the societal development [2]. For instance, some studies show that women will not disclose their HIV status to avoid being isolated from participating in the sociocultural aspect of food preparation since food is regarded as an expression of support and acceptance [21, 24], or people refuse to buy food from PLWHA [16]. Other studies show that family members of a person who died of HIV/AIDS or family members who live with PLWHA are stigmatized; therefore family members encourage PLWHA to remain silent to avoid social rejection [17, 22]. In some instances, receiving food assistance from the government also enhances a perception of difference from other members of the community since it is assumed that only PLWHA are offered such support in a community where almost everybody is poor and needs support [2, 57]. People from highly collectivist communities are more likely to be concerned with harmony and equality in the group [7]. We also need to further understand whether stigma is more or less likely to manifest itself in cultures with an extensive communal life and how stigma finds its origins in subcultural beliefs, religion, or individually conceived causal processes [6, 17, 63].

Factors that seem to mediate stigma include:

- cultural constructions, stereotypes and specific beliefs,
- access to and the role of antiretroviral therapy,
- religion,
- gender.

## 8. Cultural Constructions of HIV, Stereotypes and Specific Beliefs

The association with specific sexual behavior that is considered socially unacceptable by many people contributes to the stigma associated with HIV infection [6, 17, 22, 25]. HIV/AIDS provides an example of how illness, despite the biological characteristics of its signs and symptoms, always carries a second reality expressed in cultural images and metaphors [8–10, 17, 19, 26, 61]. Campbell et al. argue that even when ART is available and the outcome of HIV/AIDS not always fatal, the link between HIV/AIDS and bad (sexual) behavior is still a concern for PLWHA because of shame and embarrassment [6]. The second and third processes (identification and linking to immorality) apply here. Fears associated with illness, disease and sex therefore need to be viewed in their broader social and cultural context [6, 64]. To illustrate the unacceptability associated with the disease, terms such as “a long illness” or “a short illness” are deemed culturally acceptable in the obituary of someone who dies from HIV/AIDS rather than mention of the real cause of death [2, 17, 20, 27, 61]. There is also reluctance to mention the name “AIDS” while the illness is ongoing [6, 19, 26]. This process can be a way of denying HIV/AIDS or simple avoidance of explicitness or specificity as a way of coping with the serious threat of HIV/AIDS [2, 6, 13, 17, 19, 23]. Since society acts strongly against threats to tangible assets such as life, safety, property or values, it tries to sanctify the problem to protect its self-identity or reduce the negative effect [9]. The fourth process (distancing, disempowerment or control) apply here. The practice of indirection has also been noted in areas outside HIV/AIDS, such as the use of coded language in relation to certain subjects in the presence of children [6, 17]. Furthermore, mentioning HIV/AIDS can be viewed as disrespectful to the deceased [6, 61]. In some cases, acknowledging the death of a relative as due to AIDS could put the family at risk of losing the financial benefits from insurance companies since some insurance companies may not pay out benefits resulting from death due to AIDS [61]. Yet not acknowledging the cause of death to insurance companies can be viewed as a moral hazard thereby complicating the rights of dead persons who are seen as vulnerable [61]. Furthermore, in a qualitative study in Zimbabwe, denial and miss-attributions of HIV/AIDS causes (e.g., witches, unhappy ancestors, etc.) were common [27]. Less than exact terms are also used by people, including health care professionals [27], to describe HIV/AIDS to avoid insensitivity to culturally sensitive issues but not necessarily denying HIV/AIDS: for example, health care professionals in Malawi calling it ELISA disease, immunosuppression, and so forth, or lay people calling it Kaliwondewonde (slim disease), Ntengano (the disease that leads to wife and husband dying together or one after the other) [61] or other indirect descriptions [6, 17, 27]. Denial is also a way of reinforcing that HIV/AIDS is a disease of others not of the self [28] and one of the ways people as individual, group or society respond to a frightening or intolerable situation [9]. This fits with the first and second processes.

HIV/AIDS is stigmatizing because it carries many symbolic associations with danger. Attribution of contagion, incurability, immorality and punishment for sinful acts is common in many societies [6, 9, 10, 16, 19, 22, 25]. In terms of the third process, any person diagnosed with HIV is perceived to be immoral. Quam [65] argues that beliefs about AIDS as a “polluted disease” reflect people's negative evaluations of the routes through which HIV enters the body. Sexually-transmitted infections are considered to be agents of contamination or pollution in a study about HIV/AIDS prevention among African traditional healers [29]. This polluting quality of AIDS and fear of the disease are translated into stigmatizing responses such as avoidance and isolation which is where the fourth process applies (distancing). 

Self-diagnosis and self-treatment remain widespread [26, 30] owing to stigmatization. The pursuit of different therapeutic options is sometimes a result of the problematic social complexity linked to AIDS [17]. Witches and witchcraft remain an option for self-diagnosis of illnesses [23, 26] as well as for diagnosis by traditional healers [6]. This fits with the first and second processes. Commonly, people say that HIV/AIDS hides behind witchcraft since it is more culturally acceptable and it avoids personal shame [27]. People prefer to claim that they are bewitched or have (normal) tuberculosis rather than accept that they have HIV/AIDS [6, 17, 31, 32].

Stigmatization is a stereotyping response to negatively perceived characteristics of a person or group [16]. The stereotyped individuals, the context of this paper, are PLWHA; they often look, act or live differently and do not fit into the societal norms [9]. As regards to the second process, identification of PLWHA, and the third process, linking HIV/AIDS to immoral behavior, the different languages used to describe PLWHA send clear messages [3, 19]. Examples are as follows: he is a walking corpse [3] or *Kakokoolo* (scarecrow), or *Kamuyoola* (was caught in a trap) in Uganda [31] and *ashawo* (prostitute) in Nigeria. An individual's past social history may also be recalled to justify why they have AIDS [31]. PLWHA are seen as a reflection of evil and sin, leading to powerful stigma against those who have contracted the disease. In a study carried out in Tanzania, a distinction between “true” AIDS and “false” AIDS emerged, of which the former is more stigmatized and regarded as more hopeless than the latter, which is attributed to malice such as witchcraft [30]. Fear of stigma limits the efficacy of HIV-testing programs across Sub-Saharan Africa [33–35] because in most communities everyone knows sooner or later who visits test centers [31, 36]. The process of identification applies here. For some individuals, not knowing one's HIV sero-status is far preferable for being tested. For example, a study carried out in Botswana on attitudes, practices and human right concerns of routine VCT showed that 33% of the respondents did not go for voluntary counseling and testing (VCT) because a positive HIV test result will force them to stop some of their sexual practices [56] The belief is that it is better to suffer the disease quietly and hidden than to find out through HIV testing, because of the stigma associated with receiving a positive test result, in addition to the feeling that “what you don't know can't harm you” [48].

## 9. Access to and the Role of Antiretroviral Therapy

Although access to antiretroviral therapy (ART) has increased more than tenfold in low- and middle-income countries including Sub-Saharan Africa in the last six years [66], reaching the potential beneficiaries has been difficult, as the PLWHA do not identify themselves to the medical professionals [37, 56]. Individuals who were not tested for HIV demonstrated significantly greater AIDS-related stigmas ascribing greater shame, guilt and social disapproval to PLWHA [38, 56]. Studies have shown that many Sub-Saharan Africans are reluctant to disclose their HIV status even when they have already gone for VCT; moreover, those who do disclose it are selective in choosing their audience [2, 6, 7, 13, 21, 39–41, 57]. In a study carried out in the Niger Delta, Nigeria, 23% of the PLWHA respondents had not disclosed their status, while of the 77% who had disclosed their status, 22.3% disclosed it to parents, 9.7% to siblings, 27.8% to pastors, 6.3% to friends, 10.4% to family members and 23.6% to sexual partners [42]. The first and second processes (HIV/AIDS as a problem and identification) apply here. Fears of stigmatization, of victimization, of confidants spreading the word, of accusation, of infidelity, and of abandonment were all noted to be barriers to disclosure. Similar findings resulted from a study carried out in Cape Town, South Africa which showed that nearly one in four participants never talked with a friend about their HIV status [16]. Yet a different study showed that respondents who personally knew someone infected with HIV or AIDS tended to report less stereotypical and less discriminatory attitudes, fewer feelings of discomfort and less intolerant attitudes [4]. Attempts to discuss HIV make many people withdraw or feel that the discussion should be discontinued. Some of them come up with questions about the very existence of HIV [6, 56]. Evidence also shows that noninfected people intentionally distance themselves from PLWHA [67]. 

A study carried out in Ghana showed that even though PLWHA regain their strength with ART and the physically devastating effect of HIV/AIDS is tempered, they still face psychological isolation and condemnation from their family, friends and society [43] because people around them are aware of their HIV status. This is linked to the first, second and fourth processes. Another problem PLWHA face is that combinations of health-seeking strategies make it difficult to know the effectiveness of ART [44]. Many stop taking ART when the symptoms are gone and resort to traditional medications. In a study in Tanzania, many people consult both traditional and medical facilities when faced with AIDS [30]. Traditional healers are accessible, affordable and culturally acceptable [44]. They are at the grass-roots level with sufferers and can provide psychosocial support [44].

ART has also been shown to be less effective when initiated in someone with advanced disease [68] so delay in care seeking should be avoided. Apart from the medical benefits, there are also psychosocial benefits associated with seeking treatment. PLWHA who opt for an ART program can take comfort from participating because they get counselling from professionals trained to handle the psychosocial problems [41, 45]. Ironically, widespread use of ART may decrease transmission concerns [2] and increase risky behaviors [69, 70]. Patients and their partners may believe that because their viral load is undetectable and they feel so much better, the virus is absent or dead and they are incapable of transmitting HIV to others [30, 69]. Their motivation to continue condom use or other risk reduction behaviors wanes [70].

## 10. Religion

In Sub-Saharan African, many people are religious [7, 17, 22, 28]. Religious institutions have been documented as playing both supportive and detrimental roles toward PLWHA [6, 7, 21, 23, 46]. Religious leaders have the possibility as any other leaders in position to be tempted to exercise power over others [3, 6]. One of the strategies used by some churches to regain their lost moral authority is vigorously linking sexual transgressions and AIDS with sin and immorality [46, 47]. The third process (linking to immorality) applies here. AIDS has been targeted by some religious groups in order to enhance their own beliefs, morality and ideology [9, 28]. This is because sexual activity is both biological and socially-constructed behavior which reflects and can challenge strong public and private religious, cultural and political values [25, 46, 71]. The religious approach warrants stigmatizing people as “saved” or “sinner”, “pure” or “impure”, “us” or “them”, and it strengthens the broader social stratifications within which stigma flourishes [3, 7, 9, 23, 25]. The fourth process applies here, where PLWHA are distanced, disempowered or controlled. In Zambia, churches sometimes impose mandatory HIV testing before allowing marriage and individuals with HIV have been excommunicated from churches because they were deemed “sinners” [6, 61]. It is also hard to find people openly critical of the religious authorities. At the same time, many people living with HIV/AIDS express faith and religion as important in coping with HIV. Religion gives people the opportunity to accept that they are wrong but through prayer subsequently to have hope that they are forgiven and will go to a better place after death [47]; this comfort is in addition to the care and support they get, which have increased [2, 7, 21, 23, 25, 49]. Such spiritual locus-of-control beliefs are important [2]. Consultative dialog between PLWHA and religious leaders is pivotal to a successful faith-based HIV intervention [25].

## 11. Gender Issues in HIV in Africa

Stigmatization has been linked with power [6, 9, 62]. In Sub-Saharan Africa, women are traditionally expected to bear children, cook for the family [24, 28] and submit to the sexual desires of their husbands [6, 13]. Gender inequality is one of the main influential factors in women's inability to protect themselves [6, 27, 49]. Many cultures consider ignorance of sexual matters as a sign of purity, making women reluctant to seek reproductive health information and services [6, 46, 72]. Several means of transmission of HIV have been recognized, but in Africa transmission by heterosexual contact is mostly understood to be the cause [6, 7, 13, 22, 33, 60]. People believe that infection must result from indecent sexual behavior [2, 6, 17, 19, 20, 28]. The third process applies here.

Society is more intolerant of females living with HIV/AIDS than of their male counterparts [13, 16, 27, 35]. A study carried out in Kenya shows that 56% of women are commonly viewed to be targets of stigma compared with 12% of men [49]. Much of the social control over women's movements, voice and opportunities is based on the belief that they will become promiscuous if granted too much freedom and this could lead to contamination of the patriarchal lineage [27]. PLWHA have become scapegoats generally [9], but females experience an added intensity of this phenomenon, a double stigma with a bigger social disadvantage [6, 13, 27]. Women are frequently blamed as vectors of HIV transmission, although contrary to the factual process [3, 28, 31]. Most societies in Africa expect their women to be monogamous but expect men to have extramarital affairs [13, 23, 72] or to be polygamous [6, 13]. Yet a woman's monogamy does not protect her from the infection if her spouse has other sexual partners [72]. This gender aspect is even stronger in Sub-Saharan Africa because most women are dependent on their husbands for food, shelter and clothing [13, 57]. In terms of all four processes, many women refrain from testing and (if positive) would rather conceal their status. A study carried out in Ghana showed that secrecy affected women's access to treatment, and financial and emotional support from families [50]. The main reasons for not disclosing HIV status were fear of stigma and divorce [49, 51], fear of losing confidentiality [51], women's low decision-making power, communication patterns between partners and male partners' attitude to voluntary counselling and testing (VCT) [52]. In line with the female's sex role she may not insist on condom use when a partner refuses which is important for prevention and spread of HIV infection [51, 72]. Studies have shown that women who share HIV test results with their partners may experience a range of reactions from support and understanding to accusations, discrimination, physical violence and abandonment [2, 7, 21, 24, 49, 52, 53] This relates to the fourth process which is distancing, disempowerment or control. Therefore, a woman exhibiting the independence needed to protect her health risks the condemnation from her family and of the community [3]. Although the majority of the studies show female stigmatization, a study carried out in South Africa [16] showed that men were more likely than women never to have discussed AIDS with friends, more likely to have been treated differently since testing, more likely to report experiencing internalized stigma, and more likely to have suffered loss of a place to stay or job owing to AIDS. Part of the explanation for this could be the fact that men are more likely to have been working before the sickness and are primarily responsible for providing shelter [13, 16].

## 12. Consequences of HIV/AIDS Stigma and Discrimination

Stigmatization can have many consequences for PLWHA and people affected by HIV/AIDS [6, 22, 47]. Some of the consequences of HIV/AIDS stigma include lower uptake of maternity health services by women, fear of health workers getting infected and less provision of health care workers' services because they take into account the HIV status of patients [37, 40, 45]. There is also a serious implication for prevention because people do not want to go for VCT [2, 27, 56] and even those people who go for testing do not disclose their HIV status to their sexual partners owing to HIV/AIDS stigma [53] and are more likely to engage in sexually-transmitted risk behaviors and this has implications for the spread of HIV/AIDS [9, 27]. Conversely, when PLWHA are shown compassion, they are likely to take protective precautions in sexual behavior [6] and be more open about their HIV status [2]. Some spouses end up knowing of their positive HIV long after their partners are dead because they were not informed [7]. Stigma also has ongoing effects on the adherence to ART by PLWHA thus affecting their quality of life and increasing complications [2, 6, 39, 54–56]. It also leads to collective public denial of HIV/AIDS, which does not help to reduce the HIV/AIDS infection and does not help in fighting stigma [6, 17, 46]. It worsens the stress PLWHA live with, as they are forced to be silent about their status, which on its own is burdensome [2, 21, 41, 43] especially for people who need to keep their source of livelihood by keeping their jobs [2]. It affects access to social support networks either within PLWHA that will help their psychology, sharing of experiences [2, 6, 55] or from government that can offer them food supplements to improve their health or from their family as well as from their communities [21, 43, 46]. It hampers HIV-prevention and promotional efforts as people may not be willing to attend the educational programs aimed at reducing the spread of HIV/AIDS.

## 13. Conclusion

Although current literature shows that stigma in some countries, for instance, the Republic of South Africa, has started to decline over the years [13, 16], especially when it involves a close relative [33], it nevertheless is highly present [20, 22, 27, 31, 43, 55, 61]. This review was based on a systematic compilation and evaluation of literature on HIV/AIDS stigma in Sub-Saharan Africa. Detailed evaluation became possible owing to selected literature of sufficient quality and the number of publications available in Pubmed, Medscape and Psycinfo. At the same time, this implies a clear restriction. Papers on HIV/AIDS not related to stigma were not considered.

Despite the current progress of good prognostic health outcomes for HIV/AIDS, the Sub-Saharan African response still stands at a crossroads. In this paper, we contend that cultural constructions of HIV/AIDS, based on beliefs about contamination, sexuality and religion, play a crucial role and contribute to the strength of distancing reactions and discrimination in Sub-Saharan Africa by enhancing inequality. The public denial of HIV/AIDS is real and stems from a cultural undertone with a view which allows the pursuit of different treatment options, although denial on its own can be relative because it can be a way of coping with the disease while still acknowledging its existence. Denial can also be further enhanced when the PLWHA do not show some of the manifestations generally associated with HIV/AIDS by the community such as weight loss especially those on ART as people do not believe that they have HIV/AIDS even when they disclose their HIV status [2]. 

PLWHA experience stigma throughout their lifetime. Issues of stigma, discrimination and denial are still poorly understood and often marginalized within national and international programs and responses [27, 62]. Stigma prevents the delivery of effective social and medical care, enhances the number of HIV-infections and diminishes the public health effects of ART because PLWHA are not able to interact with their families and the communities which is supposed to make them feel complete and be a part of the society. 

The identification of HIV patients poses a problem, because people try to hide the disease but perceivers assume that people have HIV if they are sicker than normal. Linking HIV/AIDS to immorality is common because of religious practices and a culture of serenity. This could be remedied by instituting programs that allow people to discuss sexuality based on their cultural norms and beliefs during which some of the myths surrounding HIV/AIDS can be corrected. Putting people in touch with individual and collective strength is a key strategy to mobilizing them in such complex issues such as stigma [2, 6, 43, 46]. More qualitative studies are needed in Sub-Saharan Africa on HIV/AIDS for better understanding of stigma given that stigma regarding HIV/AIDS is rooted in local beliefs, religion and gender.

## Figures and Tables

**Table 1:** Characteristics of the empirical studies used.

Number	Author	Location number on reference list	Year of publication	Methods	Study objectives	Study population	Country
1	Greeff et al.	[2]	2008	Qualitative research design focus group discussion	To increase understanding of disclosure as a circumstance that is affected by HIV/AIDS stigma and discrimination	225	Five African countries (Tanzania, Lesotho, South Africa, Swaziland, and Malawi)

2	Campbell et al.	[6]	2007	Qualitative research In-depth interview including focused group discussion	Study on complex interplay of psychological and social forces that drive HIV/AIDS stigma	120	South Africa

3	Neville and Rubin	[7]	2007	Semistructured focused group discussion	Identity of typical targets of disclosure of positive sero-status, commonly used avenues for disclosure, motivations for disclosure and nondisclosure of sero-status	40	Kenya

4	Strebel et al.	[13]	2006	Interview and focus group discussion	Construction of gender identities and roles, how women and men understand gender-based violence and what they believe about links between gender relations and HIV/AIDS risk	78	South Africa

5	Simbayi et al.	[16]	2007	Quantitative study	Examination of internalized AIDS stigmas among PLWHA	1063	South Africa

6	Wood and Lambert	[17]	2008	Participant observation, semistructured interview, focused group discussions	Description of family and community responses to HIV/AIDS epidemic: use of indirect communication	152	South Africa

7	Muula	[18]	2005	Theoretical review	What should HIV/AIDS be called in Malawi?		Malawi

8	Uys et al.	[19]	2005	Focus group discussion	Identification of terminology used to talk about HIV/AIDS and PLWHA	261	Five African countries (Lesotho, South Africa, Malawi, Swaziland, and Tanzania)

9	Visser et al.	[20]	2009	Questionnaire	Assessment of stigmatizing attitudes among members of a community compared with perceived stigma within the community and the extent to which stigmatizing attitudes are affected by sociodemographic characteristics	1077	South Africa

10	Iwelunmor	[21]	2006	Focus group discussion	Family system responses to HIV and AIDS	204	South Africa

11	Ulasi et al.	[22]	2009	Questionnaire	Predictors of stigma and the perception of communities toward PLWHA	104	Ghana

12	Hartwig et al.	[23]	2006	Focused group discussion	A case study providing insights into how some local church leaders view HIV stigma, and changes some of them have made in their own church leadership behavior	15	Tanzania

13	Okoror et al.	[24]	2007	Focused group discussion	Role of food as an instrument in expressing and experiencing stigma used by HIV-positive women and their families	249	South Africa

14	Otolok-Tanga et al.	[25]	2007	Semistructured interview	Exploration of Uganda-based key decision-makers about the past, present and optimal future roles of faith-based organizations involved in HIV/AIDS work, including actions to promote or dissuade stigma and discrimination	150	Uganda

15	Chimwaza and Watkins	[26]	2004	Quantitative and interview	Focus on the caregivers' diagnoses of the illness of their patients, the type and duration of the care they provided, the support they received from relatives and other members of the community, and the extent to which caregiving was experienced as an emotional, physical and financial burden	15	Malawi

16	Duffy	[27]	2005	Focused group discussion, interview	Perspective on HIV-related stigma	28	Zimbabwe

17	Petros et al.	[28]	2006	Focus group discussion, interview	Exploring the concept and practice of “othering” in relation to HIV and AIDS today	418	South Africa

18	Kalichman et al.	[29]	2005	Questionnaire	Development of a brief measure of AIDS-related stigma that could be readily used in multiple settings and contexts	1371	South Africa

19	Plummer et al.	[30]	2006	Qualitative research (participant observation)	Examination of beliefs about general illness, STI and AIDS treatment practices	Participant observation	Tanzania

20	Muyinda et al.	[31]	1997	Qualitative research (in-depth interview)	Knowledge, attitudes and practices of families caring for PLWHA in relation to stigma-related conditions	127	Uganda

21	Hatchett et al.	[32]	2004	Qualitative research (interview)	Exploration of traditional and modern health-seeking in Malawi	46	Malawi

22	Thorsen et al.	[33]	2008	Qualitative research (interview, nonparticipant observation)	Potential facilitation of stigmatization through inadvertent disclosure of HIV + status via PMTCT program components and attributes	42	Malawi

23	Daniel and Oladapo	[34]	2006	Quantitative study (questionnaire)	Assessment of acceptability of prenatal HIV screening among pregnant women attending primary healthcare centres in a suburban population	333	Nigeria

24	Hutchinson and Mahlalela	[35]	2006	Quantitative (survey data using questionnaire)	Examination of patterns and determinants of use of VCT services	3374	South Africa

25	Nyblade et al.	[36]	2001	Quantitative (questionnaire), laboratory and counselling data	Assessment of self-selection in those who chose to participate in VCT and those who chose not to participate in the start-up phase of a long-term VCT program	10 950	Uganda

26	Maedot et al.	[37]	2007	Case control study	Identification of factors that determine VCT uptake among pregnant women attending ANC services	402	Ethiopia

27	Kalichman and Simbayi	[38]	2003	Quantitative research (venue intercept study)	Examination of the relationship between HIV testing, history, attitudes toward testing and AIDS stigma	500	South Africa

28	Nachega et al.	[39]	2005	Quantitative research (questionnaire)	Investigation of knowledge, attitudes, beliefs and practices of PLWHA regarding HIV/AIDS and ART in an HIV outpatient clinic	105	South Africa

29	Turan et al.	[40]	2008	Qualitative research (in-depth interview)	How HIV-related fears may affect where women deliver and the difficulties maternity workers face caring for HIV-positive women with unknown HIV status	37	Kenya

30	Orner et al.	[41]	2008	Qualitative research (focused group discussion, in-depth interview)	Exploration of perceptions and experiences of PLWHA of reproductive age in relation to HIV/AIDS care and treatment	8	South Africa

31	Akani and Erhabor	[42]	2006	Quantitative research (questionnaire)	Evaluation of rate, patterns, barriers to HIV sero-status disclosure	187	Nigeria

32	Blackstock	[43]	2005	Narrative case study	Curing stigma—the limits of antiretroviral access	1	Ghana

33	Kayombo et al.	[44]	2005	Qualitative research (interview)	Role of traditional healers in supporting orphans, how they get the orphans, the basic needs they can provide, techniques used for psychosocial support and problems encountered when taking the orphans	25	Tanzania

34	Mshana et al.	[45]	2006	Qualitative research (focused group discussion, interview)	Identify and mitigate barriers to seek ART between the stages of testing for HIV and enrolling in the new government ART program	18	Tanzania

35	Campbell et al.	[46]	2005	Qualitative research (focused group discussion, interview)	Identification of forms taken by stigma and its effects; identification of material, symbolic and contexts associated with stigmatisation of PLWHA	99	South Africa

36	Thomas	[47]	2006	Qualitative research (interview, diaries)	Exploration of how illness, the daily and long-term duties of caring amongst a sample of households in the Caprivi region	12	Namibia

37	Skinner and Mfecane	[48]	2004	Qualitative research (focused group discussion, interviews)			South Africa

38	Amuyunzu-Nyamongo et al.	[49]	2007	Quantitative (survey) and qualitative (in-depth interview)	Examination of complex relationship between gender, poverty, susceptibility to HIV and vulnerability to AIDS through drawing on the lived experiences of infected women and exploring the coping strategies they adopt; how the specific conditions of informal settlements influence these challenges and support mechanisms	390 (survey) 20 (interview)	Kenya

39	Mill	[50]	2003	Qualitative research (in-depth interview, focused group discussion)	Findings related to breaking the news of HIV infection to women and their maintenance of secrecy following diagnosis	56	Ghana

40	Antelman et al.	[51]	2001	Quantitative research	Examination of sociodemographic and behavioral factors predictive of an HIV-positive test result	1078	Tanzania

41	Maman et al.	[52]	2001	Qualitative research (interview)	Presentation of individual, relational and environmental factors that influence the decision to test for HIV-1 and to share test results with partners	62	Tanzania

42	Ndinya-Achola et al.	[53]	1995	Quantitative research	The right not to know HIV test result after being tested	5274	Kenya

43	Sanjobo et al.	[54]	2008	Qualitative research (interview, focus group discussion)	Exploration of patients' and health care professionals' perceived barriers to and facilitation of patients' adherence to ART	72	Zambia

44	Ncama et al.	[55]	2008	Quantitative research	Examination of characteristics related to social support and antiretroviral medication adherence	149	South Africa

45	Weiser et al.	[56]	2006	Quantitative research	Assessment of knowledge of and attitudes toward routine testing in Botswana with a focus on human rights concerns related to policy; factors associated with whether respondents had heard of routine testing and had positive attitudes toward the policy; the prevalence and correlations of HIV testing, barriers to and facilitation of testing and reported experiences of testing 11 months after introduction of routine testing	1268	Botswana

## References

[1] http://data.unaids.org/pub/EPIslides/2007/2007_epiupdate_en.pdf.

[2] Greeff M, Phetlhu R, Makoae LN (2008). Disclosure of HIV status: experiences and perceptions of persons living with HIV/AIDS and nurses involved in their care in Africa. *Qualitative Health Research*.

[3] Rankin WW, Brennan S, Schell E, Laviwa J, Rankin SH (2005). The stigma of being HIV-positive in Africa. *PLoS Medicine*.

[4] Gerbert B, Sumser J, Maguire BT (1991). The impact of who you know and where you live on opinions about AIDS and health care. *Social Science and Medicine*.

[5] Herek GM, Glunt EK (1988). An epidemic of stigma: public reactions to AIDS. *American Psychologist*.

[6] Campbell C, Nair Y, Maimane S, Nicholson J (2007). ‘Dying twice’: a multi-level model of the roots of AIDS stigma in two South African communities. *Journal of Health Psychology*.

[7] Miller AN, Rubin DL (2007). Factors leading to self-disclosure of a positive HIV diagnosis in Nairobi, Kenya: people living with HIV/AIDS in the Sub-Sahara. *Qualitative Health Research*.

[8] Goldin CS (1994). Stigmatization and AIDS: critical issues in public health. *Social Science and Medicine*.

[9] Gilmore N, Somerville MA (1994). Stigmatization, scapegoating and discrimination in sexually transmitted diseases: overcoming ‘them’ and ‘us’. *Social Science and Medicine*.

[10] Hardon A, Boonmongkon P, Streefland P (1995). *Applied Health Research Manual Anthropology of Health and Health Care*.

[11] Goffman E (1963). *Stigma: Notes on the Management of Spoiled Identity*.

[12] Mawar N, Saha S, Pandit A, Muhajan U (2005). The third phase of HIV pandemic: social consequences of HIV/AIDS stigma & discrimination & 
future needs. *Indian Journal of Medical Research*.

[13] Strebel A, Crawford M, Shefer T (2006). Social constructions of gender roles, gender-based violence and HIV/AIDS in two communities of the Western Cape, South Africa. *SAHARA*.

[14] Taylor B (2001). HIV, stigma and health: integration of theoretical concepts and the lived experiences of individuals. *Journal of Advanced Nursing*.

[15] Http://www.cdc.gov/mmwr/preview/mmwrhtml/mm4947a2.htm.

[16] Simbayi LC, Kalichman S, Strebel A, Cloete A, Henda N, Mqeketo A (2007). Internalized stigma, discrimination, and depression among men and women living with HIV/AIDS in Cape Town, South Africa. *Social Science and Medicine*.

[17] Wood K, Lambert H (2008). Coded talk, scripted omissions: the micropolitics of AIDS talk in South Africa. *Medical Anthropology Quarterly*.

[18] Muula AS (2005). What should HIV/AIDS be called in Malawi?. *Nursing Ethics*.

[19] Uys L, Chirwa M, Dlamini P (2005). “Eating plastic,”, “Winning the lotto,”, “Joining the www”... descriptions of HIV/AIDS in Africa. *Journal of the Association of Nurses in AIDS Care*.

[20] Visser MJ, Makin JD, Vandormael A, Sikkema KJ, Forsyth BWC (2009). HIV/AIDS stigma in a South African community. *AIDS Care*.

[21] Iwelunmor J, Airhihenbuwa CO, Okoror TA, Brown DC, Belue R (2006). Family systems and HIV/AIDS in South Africa. *International Quarterly of Community Health Education*.

[22] Ulasi CI, Preko PO, Baidoo JA (2009). HIV/AIDS-related stigma in Kumasi, Ghana. *Health and Place*.

[23] Hartwig KA, Kissioki S, Hartwig CD (2006). Church leaders confront HIV/AIDS and stigma: a case study from Tanzania. *Journal of Community and Applied Social Psychology*.

[24] Okoror TA, Airhihenbuwa CO, Zungu M, Makofani D, Brown DC, Iwelunmor J (2007). “My mother told me i must not cook anymore”—food, culture, and the context of HIV- and aids-related stigma in three communities in South Africa. *International Quarterly of Community Health Education*.

[25] Otolok-Tanga E, Atuyambe L, Murphy CK, Ringheim KE, Woldehanna S (2007). Examining the actions of faith-based organizations and their influence on HIV/AIDS-related stigma: a case study of Uganda. *African Health Sciences*.

[26] Chimwaza AF, Watkins SC (2004). Giving care to people with symptoms of AIDS in rural Sub-Saharan Africa. *AIDS Care*.

[27] Duffy L (2005). Suffering, shame, and silence: the stigma of HIV/AIDS. *Journal of the Association of Nurses in AIDS Care*.

[28] Petros G, Airhihenbuwa CO, Simbayi L, Ramlagan S, Brown B (2006). HIV/AIDS ‘othering’ in South Africa: the blame goes on. *Culture, Health and Sexuality*.

[29] Kalichman SC, Simbayi LC, Jooste S (2005). Development of a brief scale to measure AIDS-related stigma in South Africa. *AIDS and Behavior*.

[30] Plummer M, Mshana G, Wamoyi J (2006). ‘The man who believed he had AIDS was cured’: AIDS and sexually-transmitted infection treatment-seeking behaviour in rural Mwanza, Tanzania. *AIDS Care*.

[31] Muyinda H, Seeley J, Pickering H, Barton T (1997). Social aspects of AIDS-related stigma in rural Uganda. *Health and Place*.

[32] Hatchett LA, Kaponda CPN, Chihana CN (2004). Health-seeking patterns for AIDS in Malawi. *AIDS Care*.

[33] Thorsen VC, Sundby J, Martinson F (2008). Potential initiators of HIV-related stigmatization: ethical and programmatic challenges for PMTCT programs. *Developing World Bioethics*.

[34] Daniel OJ, Oladapo OT (2006). Acceptability of prenatal HIV screening at the primary care level in Nigeria. *Journal of Obstetrics and Gynaecology*.

[35] Hutchinson PL, Mahlalela X (2006). Utilization of voluntary counseling and testing services in the Eastern Cape, South Africa. *AIDS Care*.

[36] Nyblade LC, Menken J, Wawer MJ (2001). Population-based HIV testing and counseling in rural Uganda: participation and risk characteristics. *Journal of Acquired Immune Deficiency Syndromes*.

[37] Maedot P, Haile A, Lulseged S, Belachew A (2007). Determinants of vct uptake among pregnant women attending two ANC clinics in Addis Ababa City: unmatched case control study. *Ethiopian Medical Journal*.

[38] Kalichman SC, Simbayi LC (2003). HIV testing attitudes, AIDS stigma, and voluntary HIV counselling and testing in a black township in Cape Town, South Africa. *Sexually Transmitted Infections*.

[39] Nachega JB, Lehman DA, Hlatshwayo D, Mothopeng R, Chaisson RE, Karstaedt AS (2005). HIV/AIDS and antiretroviral treatment knowledge, attitudes, beliefs, and practices in HIV-infected adults in Soweto, South Africa. *Journal of Acquired Immune Deficiency Syndromes*.

[40] Turan JM, Miller S, Bukusi EA, Sande J, Cohen CR (2008). HIV/AIDS and maternity care in Kenya: how fears of stigma and discrimination affect uptake and provision of labor and delivery services. *AIDS Care*.

[41] Orner P, Cooper D, Myer L, Zweigenthal V, Bekker L-G, Moodley J (2008). Clients’ perspectives on HIV/AIDS care and treatment and reproductive health services in South Africa. *AIDS Care*.

[42] Akani CI, Erhabor O (2006). Rate, pattern and barriers of HIV serostatus disclosure in a resource-limited setting in the Niger Delta of Nigeria. *Tropical Doctor*.

[43] Blackstock O (2005). Curing stigma—the limits of antiretroviral access. *New England Journal of Medicine*.

[44] Kayombo EJ, Mbwambo ZH, Massila M (2005). Role of traditional healers in psychosocial support in caring for the orphans: a case of Dar-es Salaam City, 
Tanzania. *Journal of Ethnobiology and Ethnomedicine*.

[45] Mshana GH, Wamoyi J, Busza J (2006). Barriers to accessing antiretroviral therapy in Kisesa, Tanzania: a qualitative study of early rural referrals to the national program. *AIDS Patient Care and STDs*.

[46] Campbell C, Foulis CA, Maimane S, Sibiya Z (2005). “I have an evil child at my house”: stigma and HIV/AIDS managament in a South African community. *American Journal of Public Health*.

[47] Thomas F (2006). Stigma, fatigue and social breakdown: exploring the impacts of HIV/AIDS on patient and carer well-being in the Caprivi Region, Namibia. *Social Science and Medicine*.

[48] Skinner D, Mfecane S (2004). Stigma, discrimination and the implications for people living with HIV/AIDS in South Africa. *SAHARA*.

[49] Amuyunzu-Nyamongo M, Okeng’o L, Wagura A, Mwenzwa E (2007). Putting on a brave face: the experiences of women living with HIV and AIDS in informal settlements of Nairobi, 
Kenya. *AIDS Care*.

[50] Mill JE (2003). Shrouded in secrecy: breaking the news of HIV infection to Ghanaian women. *Journal of Transcultural Nursing*.

[51] Antelman G, Smith Fawzi MC, Kaaya S (2001). Predictors of HIV-1 serostatus disclosure: a prospective study among HIV-infected pregnant women in Dar es Salaam, Tanzania. *AIDS*.

[52] Maman S, Mbwambo J, Hogan NM, Kilonzo GP, Sweat M (2001). Women’s barriers to HIV-1 testing and disclosure: challenges for HIV-1 voluntary counselling and testing. *AIDS Care*.

[53] Ndinya-Achola J, Ambani J, Temmerman M, Piot P (1995). The right not to know HIV-test results. *Lancet*.

[54] Sanjobo N, Frich JC, Fretheim A (2008). Barriers and facilitators to patients’ adherence to antiretroviral treatment in Zambia: a qualitative study. *SAHARA*.

[55] Ncama BP, McInerney PA, Bhengu BR (2008). Social support and medication adherence in HIV disease in KwaZulu-Natal, South Africa. *International Journal of Nursing Studies*.

[56] Weiser SD, Heisler M, Leiter K (2006). Routine HIV testing in Botswana: a population-based study on attitudes, practices, and human rights concerns. *PLoS Medicine*.

[57] Http://www.kit.nl/exchange/html/2004-2_siyam_kela.asp.

[59] Link BG, Phelan JC (2001). Conceptualizing stigma. *Annual Review of Sociology*.

[60] Mosam A, Dlova NC (2006). HIV/AIDS in Sub-Saharan Africa. *Dermatologic Clinics*.

[61] Muula AS, Mfutso-Bengo JM (2005). When is public disclosure of HIV seropositivity acceptable?. *Nursing Ethics*.

[62] Parker R, Aggleton P (2003). HIV and AIDS-related stigma and discrimination: a conceptual framework and implications for action. *Social Science and Medicine*.

[63] Alonzo AA (1984). An illness behavior paradigm: a conceptual exploration of a situational-adaptation perspective. *Social Science and Medicine*.

[64] Malcolm A, Aggleton P, Bronfman M, Galvao J, Mane P, Verrall J (1998). HIV-related stigmatization and discrimination: its forms and contexts. *Critical Public Health*.

[65] Quam MD, Feldman DA (1990). The sick role, stigma and pollution: the case of AIDS. *Culture and AIDS*.

[66] http://data.unaids.org/pub/GlobalReport/2008/jc1510_2008_global_report_pp129_158_en.pdf.

[67] Stevenson MR (1991). Social distance from persons with AIDS. *Journal of Psychology & Human Sexuality*.

[68] Camp R, Huff B (2006). Antiretroviral pipeline. *What’s in the Pipeline: New Drugs, Vaccines, Microbicides, HCV and TB Therapies in Clinical Trials*.

[69] Holstad MM, DiIorio C, Magowe MK (2006). Motivating HIV positive women to adhere to antiretroviral therapy and risk reduction behavior: the KHARMA Project. *Online Journal of Issues in Nursing*.

[70] Crepaz N, Hart TA, Marks G (2004). Highly active antiretroviral therapy and sexual risk behavior: a meta-analytic review. *Journal of the American Medical Association*.

[71] Gilmore N, Jager JC, Ruitenberg EJ (1992). Human immunodeficiency virus infection and AIDS: concepts and constructs. *AIDS Impact Assessment Modelling and Scenario Analysis*.

[72] Ankrah EM, Henry K (1994). Empowering women may help retard HIV. *Network*.

